# Contribution of livestock H_2_S to total sulfur emissions in a region with intensive animal production

**DOI:** 10.1038/s41467-017-01016-2

**Published:** 2017-10-20

**Authors:** Anders Feilberg, Michael Jørgen Hansen, Dezhao Liu, Tavs Nyord

**Affiliations:** 1 Department of Engineering, Aarhus University, Hangøvej 2, 8200 Aarhus N, Denmark; 2 Present Address: Zhejiang University, College of Biosystems Engineering and Food Science, 866 Yuhangtang Road, Hangzhou, 310058 China

## Abstract

Hydrogen sulfide (H_2_S) from agricultural sources is generally not included in sulfur emission estimates even though H_2_S is the major sulfur compound emitted from livestock production. Here we show that in a country with intensive livestock production (Denmark), agriculture constitute the most important sulfur source category (~49% of all sources of sulfur dioxide), exceeding both the production industry and energy categories. The analysis is based on measurements of H_2_S using proton-transfer-reaction mass spectrometry. National emissions are obtained using ammonia as a reference pollutant with the validity of this approach documented by the high correlation of ammonia and hydrogen sulfide emissions. Finisher pig production is the most comprehensively characterized agricultural source of sulfur and is estimated to be the largest source of atmospheric sulfur in Denmark. The implication for other locations is discussed and the results imply that the understanding and modeling of atmospheric sulfate sources should include agricultural H_2_S.

## Abstract (web summary)

Hydrogen sulfide (H_2_S) from agricultural sources is generally omitted from sulfur emission estimates despite its abundance in livestock emissions. Here, the authors show that agriculture is the most important source of sulfur in Denmark using proton-transfer-reaction mass spectrometry measurements of H_2_S.

## Introduction

Emissions of hydrogen sulfide (H_2_S) contribute to the atmospheric burden of sulfur compounds, which have a major role in the formation of secondary aerosols through oxidation and conversion to aerosol sulfate^1, 2^. Aerosol sulfate is an important influence on earth radiation budget through reflection of sunlight and formation of clouds^3^, and aerosol formation poses a threat to human health^4^. In general, the contribution of H_2_S has been considered to be of minor importance compared with sulfur dioxide (SO_2_) from industry and fossil fuel combustion and dimethyl sulfide (DMS) from the marine biosphere^2, 5, 6^.

The contribution of H_2_S to atmospheric sulfur is associated with large uncertainties. Sources of atmospheric H_2_S have been reported to be: oceans, wetlands, vegetation, salt marshes/estuaries, soil, tropical forests, and volcanoes^5–8^, as well as a major anthropogenic contribution of 2.5% of SO_2_ emissions from fossil fuel combustion^2, 7^, which was estimated by Verma et al.^2^ to be the major known source of H_2_S. In a recent study on atmospheric sulfur particles, H_2_S was not included due to “the large uncertainties associated with its emission estimates”^9^.

In the atmosphere, H_2_S reacts with OH radicals with a rate constant of 4.7 × 10^−12^ cm^3 ^molecule^−1^ s^−1^ ^10^ corresponding to an estimated lifetime of 2.5 days. Gas phase reactions of H_2_S with NO_3_ radicals^10^ and ozone^11^ are too slow to be considered important, but H_2_S react rapidly (as HS^−^) with ozone in water droplets^12^, which could represent an additional H_2_S sink despite its low solubility. The ultimate end-product of gas phase H_2_S oxidation in the atmosphere is considered to be SO_2_^1^, which in turn is oxidized and ends up as aerosol sulfate. Hence, the environmental effects of H_2_S emissions can be directly compared with the effects of SO_2_ on a molar basis, but agricultural H_2_S is generally not considered as a source of secondary SO_2_ in official estimates^13, 14^. The atmospheric lifetime of SO_2_ has been estimated to be in the range of 4 to 48 h^15, 16^, and together with the OH oxidation of H_2_S this means that H_2_S can be converted to aerosol sulfate on a relatively short timescale. H_2_S is being co-emitted with ammonia and organic amines from livestock production (including waste) with ammonia being emitted by far in the largest amounts^17–19^. This is important because ammonia and amines can enhance nucleation of H_2_SO_4_^20–22^. Thus, concurrent emission of H_2_S and ammonia/amines from livestock production facilities gives rise to a plume with a strong potential for aerosol formation.

Data on emissions of H_2_S from livestock production and waste is relatively scarce, but in recent years, the application of proton-transfer-reaction mass spectrometry (PTR-MS) has provided comprehensive datasets on H_2_S emissions^17, 23, 24^ with detailed information on temporal variation.

In this work, emission of H_2_S in a region with intensive livestock production is estimated by using the concurrent emissions of ammonia (NH_3_) as a reference pollutant. Denmark is used as a relevant case due to routinely reported emissions factors of NH_3_^25–27^, and due to its high density of livestock, comparable to northwestern Germany, Netherlands, Belgium, regions in Japan, Britany in France, Catalonia in Spain, states in USA (e.g., Iowa, North Carolina, Minnesota), and other regions with intensive livestock production.

The current paper presents data from measurement campaigns carried out over 6 years from 2009 to 2015. A part of the data was extracted from studies that were aimed at investigating odor and NH_3_ emission abatement, and details concerning the locations and measurements can be found in these^17, 23, 24, 28^. The results clearly demonstrate that H_2_S from agriculture is a major source of atmospheric sulfur in Denmark and that agricultural H_2_S emissions from regions with intensive livestock production needs to be included in atmospheric sulfur budgets.

## Results

### Emission ratios of sulfur to nitrogen

The results of a series of measuring campaigns are summarized in Table 1. As can be seen, the observed mass ratios of sulfur to nitrogen (*R*_S/N_; g sulfur per g nitrogen (gS/gN)) lie within a relatively narrow range of *R*_S/N_ = 0.10–0.26 gS/gN for fattening pigs. In these calculations, only H_2_S has been considered since it is by far the most abundant sulfur compound. The only other sulfur compounds measured consistently in the ppb range are methanethiol and dimethyl sulfide, but these only constitute about 2–5% of H_2_S. A summary of organic sulfur compound concentrations together with H_2_S data is presented in Table 2. In addition to methanethiol and dimethyl sulfide, mass-to-charge ratios (*m*/*z*) corresponding to dimethyl disulfide (*m*/*z* 79 + 95) and dimethyl trisulfide (*m*/*z* 127) were detected at very low levels of typically <1 and <0.1 ppb, respectively, and contributions of other compounds at these *m*/*z* cannot be ruled out. Previously reported emissions^29–32^ of dimethyl disulfide and dimethyl trisulfide should be disregarded due to their significant formation during sampling and analysis of air-containing methanethiol when collecting samples for laboratory analysis^33, 34^.

**Table 1:** Overview of values of *R*_S/N_ obtained from the data series included in the analysis

Test site and year	Animal category	*T*_out_ (°C)^a^	H_2_S_mean_ (ppb)	NH_3mean_ (ppm)	*R*_S/N_ (gS/gN)^b^	*R* ^2^	*n*	No. of days
Site 1 (2009)	Pigs^c^	12.2	265	3.7	0.15 (0.03–0.3)	0.53	244	28
Site 2A (2010)	Pigs	13.3	373	7.3	0.18 (0.13–0.25)	0.48	168	11
Site 2B (2010)	Pigs	15.4	301	4.7	0.10 (0.06–0.13)	0.41	123	7
Site 3A (2011)	Pigs	11.7	511.3	7.4	0.24 (0.15–0.26)	0.94	1307	54
Site 3B (2011)	Pigs	11.7	520	7.5	0.25 (0.15–0.26)	0.87	1307	54
Site 3 (2012)	Pigs	8.6	420	4.3	0.23 (0.18–0.27)	0.78	396	12
Site 4 (2015)	Pigs	11.9	348	3.0	0.26 (0.16–0.36)	0.79	277	14
Site 5 (2015)	Pigs	5.6	259	3.7	0.14 (0.11–0.19)	0.55	250	7
Site 6A (Summer 2013)	Cattle	18.5	133^d^	4.6^d^	0.12 (0.04–0.25)	0.37	768	24
Site 6B (Winter 2013)	Cattle	0.4	9.1	2.4	0.009^e^ (0.007–0.012)	0.32	845	19

^a^The average outdoor temperature is included for comparison. The pig measurements were carried out in different seasons with little variation in average temperature and no significant effect of temperature on *R*_S/N_ with the exception of cattle data (see text). The average outdoor temperature in Denmark is 8.5 °C

^b^The range is included in parentheses as the 5% and 95% percentiles

^c^All pig data are based on fattening pigs (30 to ~110 kg body mass)

^d^Weighted average of room and pit concentration

^e^Data only available for room air (containing 92% of the total emission; see “Methods” section for details)

*R*_S/N_ is the mass ratio of sulfur to nitrogen emitted. Values of outdoor temperature and mean concentrations of H_2_S and NH_3_ in parts-per-billion (ppb) and parts-per-million (ppm), respectively, are included for comparison together with the coefficients of determination (*R* ^2^) for the H_2_S versus NH_3_ correlations

**Table 2:** Composition of sulfur compounds emitted from pig production facilities and the contribution of organic sulfur compounds relative to H_2_S

Location	H_2_S (ppb)	Methanethiol (ppb)	Dimethyl sulfide (ppb)	*S*_org_/H_2_S (%)
Site 1	265 ± 255	4.0 ± 1.6	4.1 ± 3.4	3.5
Site 2	353 ± 104	12.0 ± 3.4	3.0 ± 0.9	4.2
Site 3^a^	468 ± 290	7.9 ± 3.5	3.0 ± 5.2	2.3
Site 4	348 ± 154	4.6 ± 2.9	1.7 ± 0.9	1.8
Site 5	259 ± 68	3.4 ± 1.9	3.5 ± 1.1	2.7

^a^Includes 2011 and 2012 data combined

Concentration ranges are provided as one standard deviation of the mean. *S*_org_/H_2_S represents the sum of concentrations of methanethiol and dimethyl sulfide relative to the concentration of H_2_S

**Fig. 1:**
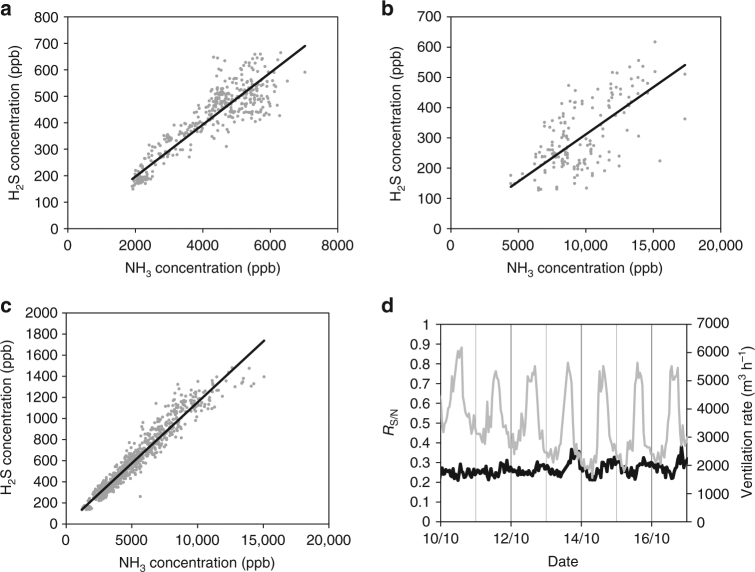
Examples of correlations between measured concentrations of H_2_S and NH_3_ in the ventilation outlet from two pig facilities. **a** Data from site 3 in 2012. **b** Data from site 2 in 2010. **c** Data from site 3 in 2011. **d** Temporal variation in the ratio of sulfur and nitrogen during 1 week of measurements (black line) together with temporal variation in ventilation rate with characteristic daytime maxima and nighttime minima (gray line). Full lines are least-squares linear regressions using a *y* axis intercept of 0. See Table 1 for further details

Generally, the emitted concentrations of H_2_S and NH_3_ are well correlated as seen in Table 1 and Fig. 1a–c. At site 3 (2011 data), measurements were performed on ventilation outlets from two identical pig units and the results were strikingly similar (Table 1). From the work reported here, it is observed that *R*_S/N_ only varies moderately indicating that differences in compound properties are of minor importance. For example, *R*_S/N_ varies surprisingly little with room ventilation rate as shown in Fig. 1d. It should be noted that ventilation rate is related to outdoor temperature to maintain a relatively constant temperature inside the pig facility, and no significant correlation of *R*_S/N_ with *T*_out_ was observed, in general. For individual data series, temperatures ranged in several cases from ~0 to ~25 °C and only in one case (site 3A–B, 2011), a significant temperature correlation was observed with lower *R*_S/N_ at higher *T*_out_ (*R* ^2^ = 0.31 and 0.66; data not shown) with a decrease in *R*_S/N_ of 2% per °C. As *R*_S/N_ was independent of *T*_out_ for all other pig data, no attempts to normalize *R*_S/N_ in relation to *T*_out_ was done. In any case, such a correction would be of little significance as the average outdoor temperature for all pig facility measurements was 11.2 °C, which is close to typical yearly average temperatures of 8–9 °C in Denmark.

For the cattle barn data, based on facilities with naturally driven ventilation, a clear difference between summer and winter is observed. During summer, *R*_S/N_ is within the range of the pig house data and the inside temperature is also close to typical inside temperatures in pig houses. However, during winter the inside temperature is significantly colder than a pig house and was mostly between 6 and 10 °C.

In addition to animal houses, H_2_S is emitted from liquid manure management as well, i.e., from manure storage and field application of manure. H_2_S emission from manure storage is generally expected to be relatively low due to stagnant liquid conditions (limiting mass transfer) and the potential for surface oxidation^35^. From previous US data^36–39^, an average *R*_S/N_ value of 0.014 for pig manure storage can be inferred. As for manure application to fields, it has been observed recently that H_2_S emissions only occur in a short time frame after application^40^, which suggests that H_2_S emission from manure application is relatively low. On the basis of data extracted from a previous study^40^, *R*_S/N_ for manure application is estimated to be ~0.001, but this ratio is associated with considerable uncertainty, as the data were obtained under controlled conditions with one type of liquid manure. Following manure application, the manure surface is largely increased compared with manure storage and under these conditions surface oxidation of H_2_S^35^ will limit emission.

## Discussion

The values of *R*_S/N_ are generally comparable with the limited literature data of simultaneous H_2_S and NH_3_ data both for pig and for cattle (Table 3). The only exception is nursery pigs for which higher ratios have been reported^39, 41^, but it remains to be confirmed if this is typical. In general, the emissions of H_2_S and NH_3_ are well correlated and occur with a relatively constant ratio for each source supporting that the values of *R*_S/N_ measured in this study can be extrapolated to regions with similar livestock production practices. For fattening pigs, *R*_S/N_ is typically within a range of 0.1–0.25 gS/gN, whereas for cattle it appears to be lower, although more data are needed to confirm this. A significant correlation of H_2_S and NH_3_ emissions has previously been observed for finisher pigs^42^.

**Table 3:** Values of *R*_S/N_ used for estimating sulfur emissions from agricultural sources

Emission source	NH_3_ emission in Denmark (2011)^a^ Gg N	*R*_S/N_ (kg S per kg N)	Data source	Additional references
Pig houses	12.8	0.19 ± 0.06^b^	This work	^18,39,41,42^
Cattle houses	10.5	0.06	This work	^41^
Pig manure storage	1.7	0.014	^37,39^	
Cattle manure storage	1.5	0.04	^53^	
Manure spreading (total)	16.8	0.001	This work	^40,51,52^
Poultry^c^	1.8	0.01	41,54	
Sheep and horses (total)	0.8	0.01	Not found^d^	
Fur (mink)^c^	5.8	0.01	Not found^d^	

^a^Data calculated based on information extracted from the Danish Normative System^27^ and from published data from the Danish Centre of Environment and Energy^26^

^b^One standard deviation included for pig data based on fattening pig values from Table 1

^c^Housing and storage combined

^d^Concurrent H_2_S and NH_3_ data not found. A conservative value of 0.01 is used, which is comparable to the lowest category (poultry). The contribution of sheep, horses and fur is estimated to be 2% of the total agricultural sources of H_2_S

*R*_S/N_ is the mass ratio of sulfur to nitrogen emitted from the source categories

For cattle, much lower *R*_S/N_-values are observed at winter due to low H_2_S concentrations, which indicate that production of H_2_S from, e.g., sulfate reduction is strongly reduced at low temperature. Mass transfer rates of H_2_S and NH_3_ are not expected to be very differently influenced by temperature based on their diffusion coefficients and their enthalpy of liquid-to-air transfer^43^. The indoor temperature in pig buildings is typically controlled at 18–22 °C due to the mechanically driven ventilation and therefore much more constant throughout the year, than a cattle barn with natural ventilation.

In livestock facilities, both H_2_S and NH_3_ are primarily emitted from the liquid waste typically collected in manure pits under a slatted floor on which the animals reside^23^. Despite this common source, some variation in the ratio of H_2_S to NH_3_ would be expected for the following reasons: variation in pH of the slurry has opposite effects on the two compounds, as H_2_S is a weak acid (p*K*_a_ at 298 K is 7), whereas NH_3_ is a base (p*K*_a_ of NH_4_^+^ at 298 K is 9.25); variation in air turbulence above the emitting slurry surface is expected to affect NH_3_ emission to a higher degree than H_2_S emission, as mass transfer of NH_3_ is mainly governed by air-side resistance^43^; H_2_S emission is predominantly limited by liquid turbulence needed to increase transport of bulk liquid H_2_S to the surface^43^. On the other hand, both H_2_S and NH_3_ originate mainly from protein in the feed. Nitrogen and sulfur are mainly excreted as urea and sulfate^44^, which are converted to H_2_S and NH_3_ in the anaerobic manure slurry by ureolytic and sulfate-reducing bacteria. The relatively consistent values of *R*_S/N_ and correlations of emissions suggest that the common source of H_2_S and NH_3_ outweighs the differences in compound characteristics.

**Fig. 2:**
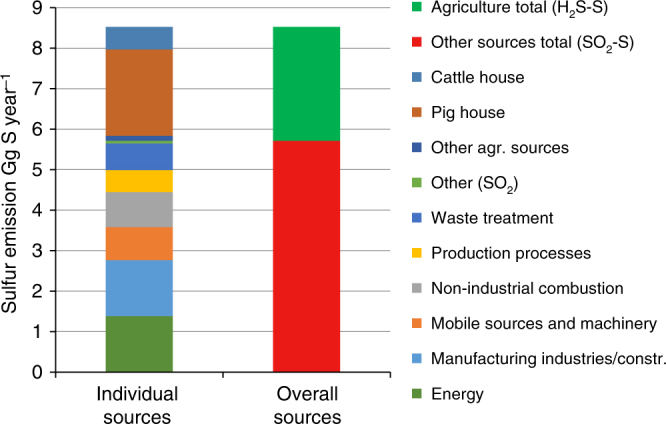
Estimated sulfur emissions for agricultural and non-agricultural sources. Sulfur is emitted as H_2_S and SO_2_ from agricultural and non-agricultural sources, respectively, and is reported in gigagrams sulfur per year. Data for non-agricultural sources (SO_2_-S) is extracted from CEIP^13^. Other (SO_2_) includes road transport, solvent use, and agricultural SO_2._ Other Agricultural Sources include slurry application (all categories) as well as fur (mink), poultry, sheep, and horses. Sulfur emissions from agricultural sources (H_2_S-S) are estimated based on values of *R*_S/N_ measured as a part of this study or estimated based on available published data (Tables 1 and 3)

For Denmark, detailed emission inventories for agriculture as well as other sectors have been available for a number of years. NH_3_ emission inventories have been routinely updated by the Danish Centre for Environment and Energy as part of the unique Danish normative system^25–27^. This provides a strong basis for using NH_3_ as a reference pollutant, which in combination with measured and estimated values of *R*_S/N_ can provide the best available estimate of H_2_S emissions from agriculture in Denmark. The result of this is provided in Fig. 2 using 2014 as a reference year based on officially reported inventories^13^. The total agricultural emission of sulfur (as H_2_S) in Denmark is estimated to be 2.8 Gg S year^−1^. Emission estimates for agricultural sources are compared with reported sulfur emission estimates from known sources^13, 45^.

It is evident that livestock houses represent a significant source of atmospheric sulfur in Denmark and although uncertainties still remain, the agricultural contribution to sulfur emissions need to be accounted for. Pig housing is estimated to be the largest single source of atmospheric sulfur in Denmark with an emission of 2.13 Gg H_2_S-S year^−1^, which is higher than, e.g., the energy sector (1.38 Gg SO_2_-S year^−1^) or manufacturing industries (1.38 Gg SO_2_-S year^−1^). Fattening pigs is the agricultural source that is by far best characterized with consistent data. This livestock category is responsible for 68% of NH_3_ emissions in Denmark and the equivalent H_2_S contribution is estimated to be 76% with the notion that more data for sows and weaners are needed. More data for cattle production is needed, especially since a strong temperature variation is indicated by the data. Emissions from other agricultural sources are much more uncertain, but their combined contribution is estimated to be relatively small.

Uncertainties in the agricultural H_2_S emission estimates are still expected to be considerable due to variation in farming practices, farm designs, manure handling systems, and feeding. Uncertainties in the reference NH_3_ emission estimates as well as the variation in observed *R*_S/N_ ratios (Table 1) influence these uncertainties. According to Mikkelsen et al.^26^ uncertainties in NH_3_ emission estimates for livestock buildings including pig houses are assessed to be 25%. Together with the variability in *R*_S/N_ of pig houses (0.19 ± 0.06; Table 3) of 32%, this gives an uncertainty of 41% by error propagation. For all other H_2_S source categories, much higher uncertainties are expected and more data is needed. However, pig houses are estimated to account for 76% of H_2_S emissions from agriculture and if assuming that the uncertainty for all other sources is close to 100%, a propagated uncertainty (corresponding to one standard deviation) in total agricultural emissions of 40% is estimated. More knowledge about variability for different sources and climatic conditions is needed to verify this uncertainty and to clarify the variation in *R*_S/N_.

The results presented here using Denmark as a case are expected to be general for similar animal production practices occur in other countries with intensive livestock production (Europe as well as regions in North America and Asia). The importance of H_2_S-S relative to SO_2_-S will of course depend on local conditions such as industrial production and fossil fuel combustion. To compare the results for Denmark with other locations, preliminary estimates based on *R*_S/N_ are compared with the Danish data in Table 4 for two cases, the Netherlands and North Carolina USA, based on 2014 data. These cases were selected as both are home to intensive livestock production and since relatively detailed emission data for NH_3_ has been published^46, 47^, which allows for application of the specific values of *R*_S/N_. The importance of agricultural H_2_S-S relative to SO_2_-S is 19% in North Carolina, 29% in the Netherlands, and 49% in Denmark. In all three cases it is clear that agricultural H_2_S is an important source of atmospheric sulfur that needs to be taken into account. In Denmark, a relatively high-livestock density together with low fossil fuel consumption gives rise to the highest influence of H_2_S. In the Netherlands, combustion in energy and transformation industries is a relatively more important source compared with Denmark and contributes 64% of SO_2_-S. However, agricultural H_2_S emission is comparable to combustion in manufacturing industry in importance for the Netherlands and exceeds by far all other sources.

**Table 4:** Comparison of sulfur emissions

Sources (DK and NL)	DK	NL	Sources (NC)	NC
SO_2_-S^13^:			SO_2_-S^14^:	
Combustion in energy and transformation industries	1.38	9.30	Fires	0.65
Non-industrial combustion plants (stationary sources)	0.86	0.28	Fuel combustion—comm/institutional	1.80
Combustion in manufacturing industry (stationary sources)	1.38	4.34	Fuel combustion—electricity generation (98% coal combustion)	23.56
Production processes (stationary sources)	0.54	0.41	Fuel combustion—industrial boilers	3.63
Solvent use and other product use	0.02	0.00	Fuel combustion—residential	0.51
Road transport	0.04	0.09	Industrial processes	4.71
Other mobile sources and machinery	0.82	0.12	Mobile sources	0.58
Waste treatment and disposal	0.66	0.002	Waste disposal	0.11
Agriculture (fossil fuel)	0.01	0.00		
Total SO_2_-S	5.72	14.54	Total SO_2_-S	36.8
H_2_S-S:			H_2_S-S:	
Pig houses	2.13	2.95	Pig houses	5.73
Cattle houses	0.55	1.05	Cattle houses	0.15
Other agricultural sources^a^	0.12	0.19	Other agricultural sources^a^	1.01
Total agricultural emissions	2.81	4.19	Total agricultural emissions	6.90

^a^Waste storage, manure application, minor animal categories

Reported emissions of sulfur (SO_2_-S) from known sources together with estimated agricultural emissions of sulfur (H_2_S-S) in Gg per year for Denmark (DK), the Netherlands (NL) and the state of North Carolina (NC). Data is calculated for 2014 based on *R*_S/N_ from the current study and reported emissions of NH_3_

For North Carolina, the dominant source of atmospheric sulfur is coal combustion, but it should be noted that the strength of this source is rapidly declining and, for example, decreased from 41.3 Gg SO_2_-S in 2011 to 23 Gg SO_2_-S in 2014^14^. According to Table 4, the second largest source of sulfur in North Carolina is agriculture (emitted as H_2_S) exceeding, e.g., industrial processes, fuel combustion in industrial boilers, and (by far) mobile sources. A previous estimate of agricultural H_2_S emissions in North Carolina was attempted by Rumsey et al.^29^ based on measurements at a single finisher pig facility. The statewide H_2_S emission was estimated to be 1.2 Gg year^−1^, which is considerably lower than the emission estimate in Table 4. No NH_3_ data were provided for comparison, but measurements of both sulfur compounds and NH_3_ were earlier performed at the same facility under similar conditions^42^. The livestock facility investigated^29, 42^ is characterized by weekly discharges of manure in the housing system and this management practice will influence emissions. The NH_3_ emission was 1.09 kg NH_3_ animal^−1^ year^−1^ ^42^ (yearly average based on measurements in four seasons), whereas a value reported in a US meta-study for finisher pig production was 4.89 kg year^−1^ animal^−1^ (3.95 kg year^−1^ animal^−1^ for all pig categories). This shows that the specific facility used in the studies by both Blunden et al.^42^ and Rumsey et al.^29^ is not typical and that results from this facility should not be directly scaled by number of animals to achieve a statewide emission. Another factor contributing to the relatively low H_2_S emission estimate achieved by Rumsey et al.^29^ is that weekly manure discharge in comparison to other manure management strategies is expected to influence H_2_S emissions to a larger degree than NH_3_ emissions^39, 48^. Even though relatively low H_2_S emissions are expected from facilities with frequent manure discharge, the *R*_S/N_-values extracted from Blunden et al.^42^ are actually comparable to the values in Table 3 with the exception of summer conditions, which gives a low-value hinting to a potential influence of temperature. North Carolina summer temperatures are relatively high compared with, e.g., Denmark.

In general, the analysis presented here shows clearly that agricultural H_2_S is an important source of atmospheric sulfur and, hence, an important precursor for aerosol sulfate in the atmosphere. The data presented here is dominated by finisher pig production, which also appear to be the most important source. Other sources should be investigated more in depth and further data on, e.g., geographical distribution, the effects of temperature and the influence of management are needed to clarify further the significance of H_2_S emission from agriculture.

## Methods

### Measurement locations

Emissions from pig farms have been measured at five different locations: An experimental pig production facility run under standard production conditions but with small pen sizes (site 1), a commercial full scale pig production facility (site 2), an experimental pig production facility run under standard production conditions with more typical pen sizes (site 3), and additional commercial full scale pig production facilities (site 4 and 5). At locations 2, 3, and 4, the data were obtained as part of testing of air scrubbers to treat emissions, but only untreated emission data are included here. At location 5, the data were obtained as part of testing manure treatment and only untreated emissions are included. No attempts were made to standardize production conditions, but all pig facilities are operated with typical feeding strategies (dry feeding ad libitum) and ventilation systems. Ventilation rate is controlled to maintain inside temperatures of 18–22 °C. All pig facilities were equipped with shallow manure pits (50–60 cm deep) used in all Danish pig facilities. These are discharged to the outside storage facility when full, typically at intervals of 5–6 weeks. Air exchange rates were obtained by using calibrated measuring fans (Fancom, the Netherlands) or a calibrated pressure difference method (Dynamic Air, SKOV, Denmark).

All emission data obtained for pig production were achieved by sampling exhaust air in the ventilation duct (outlet) of the pig facilities. Heated sampling lines (40–50 °C) were used to draw air samples to the instruments to minimize sampling-line adsorption. Sampling time varied from 10 to 20 min in each cycle. Background measurements were carried out in each measurement cycle using ambient air filtered with activated charcoal (Supelpure HC, Bellefonte, PA) and these were subtracted from the sample data.

One cattle farm (site 6) is included and measurements were done during both summertime and wintertime. This cattle farm was equipped with hybrid ventilation as detailed by Rong et al.^49^ Hybrid ventilation is not typical of cattle barns but since the majority of the air exchange takes place in the naturally ventilated animal room, it is believed to be comparable to typical cattle barns. Air exchange in the naturally ventilated room was estimated by the standard method using CO_2_ production from the animals as a naturally occurring tracer^50^. For the winter measurements, only data for the room air content of H_2_S and NH_3_ was obtained. However, this was estimated to contain 92% of the emission in the summer and is assumed to have contained most of the emission in winter as well, although presumably a lower fraction than in summer. To use wintertime data, it was assumed that values of *R*_S/N_ in the room and in the pit were comparable.

### Measurements by PTR-MS

PTR-MS was used to monitor H_2_S concentrations as well as concentrations of ammonia (NH_3_). A high-sensitivity quadropole PTR-MS (Ionicon, Austria) was used in all investigations. The PTR-MS was run at standard drift tube conditions with inlet and drift tube at 60–75 °C, drift tube pressure of 2.1–2.2 mbar, and a drift voltage of 600 V. This resulted in electrical charge-to-molecular densities in the range of 130–140 Townsend. The PTR-MS response to H_2_S (*m*/*z* 35) was calibrated based on certified gas cylinders and by taking into account the dependency of the response to the sample air humidity as described previously^17, 24^. Other sulfur compounds, methanethiol (*m*/*z* 49) and dimethyl sulfide (*m*/*z* 63), were also measured by PTR-MS. The measurement of volatile sulfur compounds by PTR-MS (including calibration) is described in detail in previous papers^17, 24^. Dimethyl disulfide was measured by detection of *m*/*z* 95 (M + 1) and *m*/*z* 79 (fragment ion; loss of –CH_3_). Owing to the contribution of phenol to *m*/*z* 95, an upper limit of dimethyl disulfide was estimated on *m*/*z* 79. Dimethyl trisulfide was measured by detection of *m*/*z* 127.

An upper limit of *R*_S/N_ for untreated manure application was estimated based on laboratory experiments with soil and manure in dynamic flux chambers. Details of the setup has been reported previously^40^. A concentration-time profile based on PTR-MS data was reconstructed and used with NH_3_ data for this purpose, but the rapid cease in H_2_S emissions contributed to a significant uncertainty. Reliable *R*_S/N_ data for manure application has not been obtained by other experiments, but field data confirm that H_2_S emission is low and ceases rapidly after application^51, 52^.

NH_3_ was at all locations measured by PTR-MS using *m*/*z* 18 as the NH_3_ signal and subtracting instrumental background at this mass-to-charge ratio. The instrumental background is relatively high for *m*/*z* 18 due to ions formed in the ion source. For site 2 and 4, additional measurements were performed by photoacoustic IR detection using a factory-calibrated Innova photoacoustic analyzer 1412 and a multi-point sampler1309 (Lumasense Technology A/S, Denmark). In general, good agreement between PTR-MS and photoacoustic measurements were observed (differences typically within 10–20%) as has been reported previously^40^.

### Data availability

The datasets analyzed during the current study are available from the corresponding author on reasonable request.

## Electronic supplementary material

Peer Review File

## References

[1] Pitts Jr J. N. & Finlayson-Pitots, B. J. *Chemistry of the Upper and Lower Atmosphere* (Academic Press, San Diego, 2000).

[2] Verma S (2007). Modeling and analysis of aerosol processes in an interactive chemistry general circulation model. J. Geophys. Res. Atmos..

[3] Fiore AM (2012). Global air quality and climate. Chem. Soc. Rev..

[4] Pope CA, Dockery DW (2006). Health effects of fine particulate air pollution: Lines that connect. J. Air. Waste. Manage. Assoc..

[5] Seinfeld, J. H. & Pandis, S. N. *Atmospheric Chemistry and Physics: From Air Pollution to Climate Change* (John Wiley & Sons, New York, 1997).

[6] Watts SF (2000). The mass budgets of carbonyl sulfide, dimethyl sulfide, carbon disulfide and hydrogen sulfide. Atmos. Environ..

[7] Aneja VP (2008). Workshop on agricultural air quality: state of the science. Atmos. Environ..

[8] Kinsela AS (2011). Field-based measurements of sulfur gas emissions from an agricultural coastal acid sulfate soil, Eastern Australia. Soil Res..

[9] Perraud V (2015). The future of airborne sulfur-containing particles in the absence of fossil fuel sulfur dioxide emissions. Proc. Natl Acad. Sci. USA.

[10] Atkinson R (2004). Evaluated kinetic and photochemical data for atmospheric chemistry: Volume I - gas phase reactions of O-x, HOx, NOx and SOx species. Atmos. Chem. Phys..

[11] DeMore WB (1997). Chemical kinetics and photochemical data for use in stratospheric modeling. Evaluation number 12. JPL Publ..

[12] Hoigne J, Bader H, Haag WR, Staehelin J (1985). Rate constants of reactions of ozone with organic and inorganic-compounds in water 3. inorganic-compounds and radicals. Water. Res..

[13] CEIP. *Centre on Emission Inventories and Projections: Officially Reported Emission Data*. http://www.ceip.at/ms/ceip_home1/ceip_home/webdab_emepdatabase/reported_emissiondata/ (2017).

[14] US-EPA. *2014 National Emissions Inventory* (*NEI*) *Data*. https://www.epa.gov/air-emissions-inventories/2014-national-emissions-inventory-nei-data (2017).

[15] Fioletov VE, McLinden CA, Krotkov N, Li C (2015). Lifetimes and emissions of SO_2_ from point sources estimated from OMI. Geophys. Res. Lett..

[16] Lee C (2011). SO_2_ emissions and lifetimes: estimates from inverse modeling using in situ and global, space-based (SCIAMACHY and OMI) observations. J. Geophys. Res. Atmos..

[17] Feilberg A, Liu D, Adamsen APS, Hansen MJ, Jonassen KEN (2010). Odorant emissions from intensive pig production measured by online proton-transfer-reaction mass spectrometry. Environ. Sci. Technol..

[18] Kim KY (2007). Sulfuric odorous compounds emitted from pig-feeding operations. Atmos. Environ..

[19] Blunden J, Aneja VP (2008). Characterizing ammonia and hydrogen sulfide emissions from a swine waste treatment lagoon in North Carolina. Atmos. Environ..

[20] Ball SM, Hanson DR, Eisele FL, McMurry PH (1999). Laboratory studies of particle nucleation: Initial results for H2SO4, H2O, and NH3 vapors. J. Geophys. Res. Atmos..

[21] Almeida J (2013). Molecular understanding of sulphuric acid-amine particle nucleation in the atmosphere. Nature.

[22] Zhang RY, Khalizov A, Wang L, Hu M, Xu W (2012). Nucleation and growth of nanoparticles in the atmosphere. Chem. Rev..

[23] Liu D, Feilberg A, Adamsen APS, Jonassen KEN (2011). The effect of slurry treatment including ozonation on odorant reduction measured by in-situ PTR-MS. Atmos. Environ..

[24] Hansen MJ, Liu D, Guldberg LB, Feilberg A (2012). Application of proton-transfer-reaction mass spectrometry to the assessment of odorant removal in a biological air cleaner for pig production. J. Agric. Food. Chem..

[25] Mikkelsen, M. H., Albrektsen, R. & Gyldenkærne, S. *Danish Emission Inventory for Agriculture*. NERI Technical Report No. 810. Inventories 1985-2009. Report No. 810 (National Research Institute, Aarhus University, Aarhus, 2011).

[26] Mikkelsen, M. H., Albrektsen, R. & Gyldenkærne, S. *Danish Emission Inventory for Agriculture. Scientific Report from DCE—Danish Centre for Environment and Energy No. 108*. Inventories 1985-2011. Report No. 108 (Danish Centre for Environment and Energy, Aarhus, 2014).

[27] Poulsen, H. D., Lund, P., Sehested, J., Hutchings, N. J. & Sommer, S. G. in *12th Ramiran International Conference: Technology for Recycling of Manure and Organic Residues in a Whole-Farm Persepctive* (ed. Petersen, S. O.) 105–107 (Aarhus University, Aarhus, 2006).

[28] Liu D, Lokke MM, Riis AL, Mortensen K, Feilberg A (2014). Evaluation of clay aggregate biotrickling filters for treatment of gaseous emissions from intensive pig production. J. Environ. Manage..

[29] Rumsey IC, Aneja VP, Lonneman WA (2014). Characterizing reduced sulfur compounds emissions from a swine concentrated animal feeding operation. Atmos. Environ..

[30] Filipy J, Rumburg B, Mount G, Westberg H, Lamb B (2006). Identification and quantification of volatile organic compounds from a dairy. Atmos. Environ..

[31] Hobbs PJ, Webb J, Mottram TT, Grant B, Misselbrook TM (2004). Emissions of volatile organic compounds originating from UK livestock agriculture. J. Sci. Food. Agric..

[32] Parker DB (2013). Odorous VOC emission following land application of swine manure slurry. Atmos. Environ..

[33] Andersen KB, Hansen MJ, Feilberg A (2012). Minimisation of artefact formation of dimethyl disulphide during sampling and analysis of methanethiol in air using solid sorbent materials. J. Chromatogr. A..

[34] Lestremau F, Andersson FAT, Desauziers V (2004). Investigation of artefact formation during analysis of volatile sulphur compounds using solid phase microextraction (SPME). Chromatographia.

[35] Nielsen DA, Nielsen LP, Schramm A, Revsbech NP (2010). Oxygen distribution and potential ammonia oxidation in floating, liquid manure crusts. J. Environ. Qual..

[36] Trabue S, Kerr B, Bearson B, Ziemer C (2011). Swine odor analyzed by odor panels and chemical techniques. J. Environ. Qual..

[37] Trabue S, Kerr B (2014). Emissions of greenhouse gases, ammonia, and hydrogen sulfide from pigs fed standard diets and diets supplemented with dried distillers grains with solubles. J. Environ. Qual..

[38] Trabue S, Kerr B, Scoggin K (2016). Odor and odorous compound emissions from manure of swine fed standard and dried distillers grains with soluble supplemented diets. J. Environ. Qual..

[39] Liu Z, Powers W, Murphy J, Maghirang R (2014). Ammonia and hydrogen sulfide emissions from swine production facilities in North America: a meta-analysis. J. Anim. Sci..

[40] Feilberg A, Bildsoe P, Nyord T (2015). Application of PTR-MS for measuring odorant emissions from soil application of manure slurry. Sensors.

[41] Zhu J, Jacobson L, Schmidt D, Nicolai R (2000). Daily variations in odor and gas emissions from animal facilities. Appl. Eng. Agric..

[42] Blunden J, Aneja VP, Westerman PW (2008). Measurement and analysis of ammonia and hydrogen sulfide emissions from a mechanically ventilated swine confinement building in North Carolina. Atmos. Environ..

[43] Sommer, S. G. & Feilberg, A. in *Animal Manure Recycling: Treatment and Management* (eds Sommer, S. G., Christensen, M. L., Schmidt, T. & Jensen, L. S.) 131–151 (Wiley & Sons Ltd., Chichester, 2013).

[44] Le PD, Aarnink AJA, Ogink NWM, Becker PM, Verstegen MWA (2005). Odour from animal production facilities: its relationship to diet. Nutr. Res. Rev..

[45] Nielsen, O. K. *et al*. *Annual Danish Informative Inventory Report to UNECE. Emission Inventories from the Base Year of the Protocols to Year 2013* (Aarhus University, DCE, Aarhus, 2015).

[46] Velthof GL (2012). A model for inventory of ammonia emissions from agriculture in the Netherlands. Atmos. Environ..

[47] Stephen K, Aneja VP (2008). Trends in agricultural ammonia emissions and ammonium concentrations in precipitation over the Southeast and Midwest United States. Atmos. Environ..

[48] Lim TT, Heber AJ, Ni JQ, Kendall DC, Richert BT (2004). Effects of manure removal strategies on odor and gas emissions from swine finishing. Trans. ASAE.

[49] Rong L, Liu D, Pedersen EF, Zhang G (2014). Effect of climate parameters on air exchange rate and ammonia and methane emissions from a hybrid ventilated dairy cow building. Energy Build..

[50] Mendes LB (2015). Spatial variability of mixing ratios of ammonia and tracer gases in a naturally ventilated dairy cow barn. Biosyst. Eng..

[51] Feilberg A, Nyord T, Hansen MN, Lindholst S (2011). Chemical evaluation of odor reduction by soil injection of animal manure. J. Environ. Qual..

[52] Feilberg, A., Liu, D. Z. & Nyord, T. in *Nose 2014: 4th International Conference on Environmental Odour Monitoring and Control* Vol. 40: Chemical Engineering Transactions (ed. DelRosso, R.) 55–60 (NOSE, Venice, 2014).

[53] Maasikmets M, Teinemaa E, Kaasik A, Kimmel V (2015). Measurement and analysis of ammonia, hydrogen sulphide and odour emissions from the cattle farming in Estonia. Biosyst. Eng..

[54] Ni JQ (2012). Characteristics of ammonia, hydrogen sulfide, carbon dioxide, and particulate matter concentrations in high-rise and manure-belt layer hen houses. Atmos. Environ..

